# Bringing climate health conversations to frontline clinics: A qualitative post-intervention assessment of utilization of the Climate Resilience for Frontline Clinics Toolkit

**DOI:** 10.1016/j.joclim.2025.100444

**Published:** 2025-05-01

**Authors:** Yi-Ting Hana Lee, Mingyue Ma, Caroline Sarpy, Casey Dai, Jinia Sarkar, Chelsea Heberlein, Theodore Miles, Caleb J. Dresser

**Affiliations:** a Department of Epidemiology, Harvard T.H. Chan School of Public Health, Boston, MA, USA; b Department of Environmental Health, Harvard T.H. Chan School of Public Health, Boston, MA, USA; c Department of Global Health, Harvard T.H. Chan School of Public Health, Boston, MA, USA; d Americares Foundation, Stamford, CT, USA; e Department of Emergency Medicine, Beth Israel Deaconess Medical Center, Boston, MA, USA

**Keywords:** Climate resilience, Frontline clinics

## Abstract

**Introduction:**

Community health centers and clinics are on the frontlines of climate change and adverse health effects, providing essential care to millions of low-income, uninsured, and underinsured populations across the country. The Climate Resilience for Frontline Clinics Toolkit (“the toolkit”) was developed to support frontline clinicians in preparing for climate-related health risks. The objectives of this study were to assess the utilization and challenges in the implementation of the toolkit in real-world clinic settings and to guide further development of clinic-based risk reduction resources.

**Methods:**

A qualitative, semi-structured interview and post-intervention assessment approach was used to interview 28 clinicians and staff from 15 clinics across six states.

**Results:**

Participants generally found the toolkit valuable, noting that it addressed an unmet need by providing actionable information on climate health risks in resource-constrained settings. However, challenges included information overload, the complexity of patient-facing materials, and concerns about literacy barriers. Many participants felt that the toolkit could benefit from more concise and visually supported materials, as well as adjustments to better align with patient literacy levels.

**Discussion:**

These findings highlight the importance of tailoring resources to the specific needs of frontline clinics and their patient populations. Future research should examine the long-term impacts of integrating such resources on patient behaviors and health outcomes and explore strategies for integrating climate resilience into routine clinical care.

## 1 Introduction

Climate change is leading to escalating health risks, creating a need for risk reduction approaches that can be deployed in clinical settings. Exposure to hazards including dangerous heat, tropical cyclones, flooding, drought, and wildfires is increasing in frequency and intensity as a result of climate change [1]. Such events are detrimental to the health and well-being of populations across the United States (U.S) and around the world [2,3].

Epidemiological studies have shown that certain populations including older adults, children, members of historically marginalized groups, those of lower socioeconomic status, individuals who are pregnant or have certain medical conditions, outdoor workers, and those living in high-risk locations such as floodplains and urban heat islands are at particularly high risk of health harms from climate-responsive hazards [[4], [5], [6]]. Meeting the needs of patients who belong to one or more of these groups requires developing and deploying interventions that are focused on their needs and appropriate to the settings in which they receive care. In the U.S., free and charitable clinics, federally qualified health centers, and other community-based clinics (“frontline clinics”) provide care to >30 million patients, many of whom belong to these groups [7,8]. Resources to support patient-centered, climate-smart care in frontline clinics are urgently needed [9].

However, while health harms associated with climate change are increasingly well documented, optimal approaches to address this issue in clinical settings remain unclear and are an active area of research and development. A 2021 survey of 430 clinicians and administrators found that frontline clinics were experiencing operational disruptions and impacts on patients’ health due to climate-related events [10]. A subset of 284 administrators and staff at frontline clinics reported a lack of knowledge and resources as barriers to improve clinic resilience [11]. Based on these results, the Climate Resilience for Frontline Clinics Toolkit (“the toolkit”) was developed, including resources for providers, patients, and administrators on risk reduction and health protection before, during, and after climate-responsive events including heat waves, hurricanes, floods, and wildfires [12]. The toolkit was initially co-developed with 9 clinics during 2022, and an implementation pilot was conducted in 18 frontline clinics across the U.S. during summer and fall of 2023.

The objectives of this study were to assess the utilization of the Climate Resilience for Frontline Clinics Toolkit [12] by clinicians and administrators in frontline clinics, identify implementation barriers and facilitators, and provide guidance on future efforts to deploy climate resilience resources in frontline clinics.

## 2 Methods

This study is a qualitative, interview-based, post-intervention assessment of real-world utilization of the Climate Resilience for Frontline Clinics Toolkit.

### 2.1 Research context

During Spring 2023, 18 frontline clinics from the Southern and Western regions of the U.S. began pilot implementation of the toolkit. The frontline clinics included Federally Qualified Health Centers (FQHCs) and Free and Charitable Clinics (FCCs). All clinics were in high-risk areas for at least two of the four hazards addressed in the Toolkit (heat, hurricanes, wildfires and floods). Each clinic engaged one clinician and one administrative staff member to implement the toolkit into their workflow from June through September of 2023.

### 2.2 Recruitment and participants

In Fall 2023, we contacted 36 clinicians and administrative staff members from the 18 clinics to participate in individual semi-structured interviews to understand their experiences implementing the toolkit and suggestions for improvements.

### 2.3 Interviews

We developed a standardized interview guide exploring three main assessment areas: context and impression, implementation including barriers and facilitators, and areas for improvement (Supplementary Table 1). The questions were drafted based on prior experience conducting interviews on related resources and reviewed by the study team, including a qualitative methods expert. Trained study staff conducted the interviews via remote video conference in October and November 2023. Audio recordings of all interviews were captured through the remote video conference platform and transcribed verbatim by an external transcription service. All identifiable information was removed prior to analysis. Study team members reviewed a random sample of interviews to ensure quality of transcription.

### 2.4 Analysis

The codebook was developed deductively based on the interview questions and included seven themes: language, content, dissemination, communication, facilitators, barriers and additional assistance/resources. These themes were used as the parent codes and were selected to inform areas of improvements in the future iterations. Study team members (YL, MM, CS, CD, JS and CJD) independently coded the first interview using the codebook, and met to discuss discrepancies, redefine code definitions and revise the codebook inductively. The revised codebook was used to code the 27 interview transcripts. Each transcript was double coded by rotating coding pairs (YL, MM, CS, CD, JS) to ensure consistent application of codes using Dedoose Version 9.2.12 qualitative analysis software. There were frequent discussions among the coders to compare and reconcile differences through consensus. When necessary, modifications to the codebook were made. Decision trails were documented to ensure that interpretations were supported by data. This process continued iteratively until all the transcripts had been double coded with the final codebook and all discrepant codes between each of the coding pairs had been discussed and agreement reached. We performed deductive qualitative content analysis in line with Patton [13,14] and the process was guided by a qualitative methods expert. The study was reviewed by the Institutional Review Board (IRB) and determined to not be human subjects research (Protocol: IRB23–1194). All participants received a consent form prior to the interview and provided verbal consent before the interview started.

## 3 Results

We invited 36 individuals to participate in semi-structured interviews: 1 clinician and 1 administrator from each of the 18 participating clinics. Of the 36 people contacted, 28 (15 clinicians and 13 administrative staff), representing 15 clinics in 6 states, agreed to participate in an interview. We report themes related to the facilitators and barriers to implementation of the toolkit, lessons learned for implemention in clinical settings, and areas of improvement.

### 3.1 Facilitators

#### 3.1.1 Content

Many toolkit users reported that the content was useful, topical, easy to understand and increased their awareness:

“Great information […] there's a lot of information on there that I wouldn't have thought to put together […] whether it's by a provider or for a patient. You can tell a lot of thought [went] into it, a lot of good information, valuable information that we were able to share.” (Clinic Staff)

Some clinicians appreciated the social determinants of health framing, as this aligned with existing models for providing comprehensive care:

“[…] our patient population has a lot of social determinants of health needs, housing needs, transportation needs […] those needs are intensified in the face of heat, in the face of hurricane, in the face of flooding was very surprising […] this conversation started in the extreme weather and climate change topic area, and it kind of drifts over into social determinants of health. Having access to appropriate air conditioning, having access to a second place to go, knowing where that place is, having the transportation to get there if you need to evacuate or relocate […]continues to highlight that disparity for our patient population and the needs that are there.” (Clinician)

Several clinicians noted that the toolkit helped initiate conversations with patients about climate-related hazards, such as discussing preparation for dangerous heat:

“I think it's going to be always the need to educate patients and have them kind of aware of what they need to do to make sure they're taking care of themselves … so it's kind of a proactive kind of situation using them.” (Clinician)

Administrative staff reported that the information on improving clinic climate preparedness improved operational procedures:

“[…] the operational checklist was probably the most valuable because that is a pretty solid starting point and structure to work with […] If you had to start from scratch, it'd be like, yeah, I don't even know where to start.” (Clinic Staff)

#### 3.1.2 Communication

Many clinicians felt the toolkit was helpful for communicating with patients. Infographics and other visuals such as a urine color chart were perceived as especially useful for patients with lower literacy:

“[…] if they had diabetes or anything, we would give them the printouts. The dehydration pictures […] using the infographics was very helpful. A lot of our patients are visually inclined to learn that way.” (Clinic Staff)

The toolkit also prompted proactive conversations about risks of climate events without making patients feel targeted:

“So there's an opportunity for us to say, ‘Hey, you mentioned this thing about the fact that you don't have air conditioning in your house, or you have unreliable electricity. Well, here's some things that we can talk to you about, and here's some handouts that you can have.’ And that actually is a great way for us to talk about that without making the patient feel that they're being singled out because they have substandard living situation.” (Clinic Staff)

Translation into Spanish was noted as helpful for Spanish-speaking patients by most interviewees.

As part of implementation, clinic staff were asked to join periodic meetings with other participating clinics. Several interviewees noted that the meetings supported implementation, and sometimes led to discussions about improving clinic policies and strategies around climate-related health hazards. The discussions were useful even for clinics from distant regions with differing climate hazards:

“…there was discussion about what had worked and what some were finding. And although, you know, some were in hurricane areas, some were in fire areas, […] just some of the ways they were utilizing the information, where in the process of a visit perhaps it was addressed, you know, the language, low literacy, you know, there were good ideas.” (Clinic Staff)

#### 3.1.3 Toolkit dissemination and implementation

The toolkit is publicly accessible online in a downloadable portable document format (PDF). For many clinicians, the electronic format was easy to navigate for the clinician's own use, to share and include in staff trainings, and to facilitate patient education.

"[…] I keep it on my tab on my computer, so I always have my laptop with me when I'm in the room. I'm able to bring it up, and I'm able to share it. (Clinician) However, paper printouts were also preferred for practical reasons in the case of dangerous weather and power outages when internet access is not available. They also made sharing information possible for patients with limited access to technology.

“[…] once we decided to print them out and have them as handouts, that took the one thing away that we were concerned about, which was the lack of technology for people to access the information.” (Clinic Staff)

“[…] our population also does not have the resources to read, to have technology use or knowledge to it […] So we did a lot of printouts […]” (Clinic staff)

Several providers shared that the resources were well-organized and user-friendly. A key feature was the centralization of the resources:

“I think it helped having everything […] on the same page and streamlined. It was a lot of information in one spot, but it was nice that everything was […] categorized and in the same place […] on the administrative side, as we think about what admin looks like and how do we as a facility respond to emergencies, all the information is just really nicely categorized […] That's really helpful.” (Clinic Staff)

Several clinics also incorporated the toolkit into text messaging or electronic health record platform messaging to remind patients about climate-related health hazards and steps they could take to stay safe:

“And what we did is […] send a message sharing the Toolkit every time we send the appointments out to confirm them. Oh, by the way, summer is here, and right now this is an additional resource […] when they checked in, we told them the same thing, and already the providers were kind of on board too, like, hey, did you receive this? Let's talk about it.” (Clinic Staff)

#### 3.1.4 Receptive organizational culture

Many interviewees reported that a supportive clinical environment was critical to successful implementation of the toolkit. This included existing interest in the health impacts of climate change and willingness to engage from all relevant stakeholders (e.g. clinic directors, administrative staff, clinicians and patients).

“So the fact that we've had that good support and our practice manager in our practice, our director has been so supportive […] in making sure that we have the toolkit, that it's something that we can utilize.” (Clinician)

“They (patients) were just thankful for the additional information that we provided outside of this, their normal health care. So yeah, it was pleasantly received.” (Clinic Staff)

Administrator and leadership involvement was often driven by a personal interest in climate change (e.g., having experienced extreme weather), patient vulnerabilities to climate change, and sense of urgency to act. Some clinics already had climate and health working groups in place.

“I think just seeing and feeling how hot it was, realizing… I mean, it's all over the news. And that kept it in the forefront for all of us.” (Clinician)

“…(w)e had a climate and health working group that was in place. So working as a team was certainly helpful.” (Clinician)

### 3.2 Barriers and proposed solutions

#### 3.2.1 Content

Several interviewees suggested for inclusion of other weather hazards, such as tornados and extreme cold events.

“[…E]specially with the emergency preparedness […] with hurricanes […], we have time to prepare for those. We know they're coming. With tornadoes, there's a very immediate response time that I think the toolkit could benefit from adding in the tornadoes.” (Clinic Staff)

Nevertheless, the breadth and thoroughness of the content needs to be balanced with relevance for practical use. Many interviewees reported feeling overwhelmed by the volume of content. This issue was compounded by limited time, a common constraint for providers at frontline clinics.

“[T]his is something I consider myself really interested in [… But] seeing a six-page handout is […] overwhelming with […] the other responsibilities that we have during the day […].” (Clinician)

Many clinicians emphasized the importance of ensuring that all patient-facing content in the toolkit is concise and actionable. Some providers expressed concerns of overwhelming patients with adverse social determinants of health and existing medical conditions.

“[T]here are sheets that have almost too much information. So there are some that could be […] bulleted […] to not overwhelm some patients […]. [If] you have a diabetic patient that is facing evacuation and a hurricane, perhaps having just a checklist to go along with the tool sheet […]. You can go back and read this later because it is overwhelming in the moment.” (Clinician)

#### 3.2.2 Organizational support and workflow

Most clinics expressed that communicating climate change related health risks should be an integral part of clinical practice. However, a few clinics cited skepticism among administrators and clinicians as a limitation to implementation.

“There's definitely some underutilization on our end […]. [T]here's still some tendency to think that climate change may not be impacting healthcare.” (Clinic Staff)

Furthermore, even when staff members were supportive of the program, competing responsibilities and resource constraints were barriers to implementation:

“[Climate change is] still not in the top five for our leadership, even though they all agree that it's bad […]. Our leadership has just cheered us on from the sidelines as our working group has done […] these efforts, and as our providers have tried to fit this into their lunch breaks […]. But it is a struggle […] because it is […] administrative time. It's not service provision.” (Clinic Staff).

Some suggested that short videos or presentations would help demonstrate the value of the toolkit.

“Our biggest issue has been […with getting] administrative support and being able to have a set of slides that talks about why this is important for us from a human health and patient care perspective, as well as from a financial perspective, would be really helpful.” (Clinician)

A couple interviewees mentioned integration of toolkit into electronic health record (EHR) systems to facilitate data collection for program monitoring and evaluation, and combat time constraints. One interviewee suggested that adding climate-related screening questions to their intake form would help quantify the proportion of patients unaware of climate-related hazards.

“I put a request to our [EHR system vendor] to see if we could upgrade our intake form [… to ask] simple questions like […] what type of work do you do? Are you aware of dehydration? […That way,] I [could] pull a report that […shows] these were the samples that we have for the Americares initiative. And […] we have X amount of percentage of patients that don't think about [the risk].” (Clinic Staff)

Additionally, streamlining the process for providers could reduce the need for extensive training, particularly in environments with high staff turnover.

#### 3.2.3 Patients

The patient materials were designed to be at 5th grade reading level. However, many still expressed that the patient-facing materials did not match patients’ literacy level.

“[Another problem is to…] have it at a reading level that's appropriate. [… M]aybe an elementary-age reading level because it's great information, but […] a lot of [times] if I was sitting there with a patient going over this information, I would need to be doing a lot of explanation […so] maybe bringing the reading level down […] to make it more inclusive.” (Clinic staff)

There were also concerns about language complexity and dialect usage in the Spanish version of the toolkit. One interviewee shared that a staff member who is a native Spanish speaker struggled to understand some of the words used in the translated version. However, the interviewee acknowledged that “there are multiple different dialects of Spanish in use” and “most of [their] patients were from Mexico or Central America, […] which is [probably] not the background of the folks that did the translation for [the toolkit].” (Clinician)

Many interviewees felt the separate documents with similar action items for different chronic conditions were redundant. Some interviewees recommended “[condensing] the information to where it applies to multiple comorbidities” (Staff) while others hoped to have more differentiated tip sheets tailored to specific conditions.

Interviewees expressed a strong interest in using short videos to share information with patients (e.g. on social media, via text messages, and on TV monitors in waiting rooms). One clinic has already converted information sheets into auto-playing PowerPoint presentations displayed in the waiting room, but many interviewees suggested patient-focused videos in multiple languages.

Of note, a clinical staff raised the concern that the toolkit was too “polarizing” by directly using the phrase climate change, which made some patients resistant to listening.

“[T]he information around what to do in a disaster is awesome and needed and super helpful, […but] always tying it to climate crisis is going to prevent some people from taking it as seriously as they would have if you didn't word it that way. […I]f we want to get people to use it, […] the more polarizing language we can remove from it, the better the usage […and] acceptance rate […] will be.” (Clinician)

## 4 Discussion

This qualitative post-intervention assessment provides insights to opportunities and challenges associated with implementing the toolkit in real-world, low-resource clinical settings. Of the 28 clinicians and clinic staff from 15 clinics who participated in this study, the majority felt that the toolkit provided novel and useful information on how to prepare for extreme weather events and helped initiate conversations with patients on climate-related health risks. The publicly available, centralized resources improved users’ experiences. Clinicians and staff found discussions with other clinics that were also implementing the toolkit to be valuable. Many noted that a supportive clinical environment and culture were key to successful implementation.

On the other hand, a lack of leadership buy-in and skepticism among clinicians, patients, and staff hindered implementation at some sites. Another challenge was achieving an appropriate balance between comprehensiveness and conciseness when presenting complex topics in a time-limited environment. Lastly, while patient-facing materials were written at 5th grade reading level and translated into Spanish, many participants felt that language complexity was still beyond their patients’ literacy level. Revising the language to match patients’ literacy level, incorporating visual aids and including short videos could increase effectiveness of communication with patients.

While a resource focused on climate-related health risks is new in the healthcare setting, similar tools have been developed for other health issues. Findings from this study are corroborated by the existing literature. For example, studies have found that infographics, a medium with short text and visual aids, have been effective in motivating smoking cessation among patients with chronic obstructive pulmonary disease (COPD), raising awareness for traffic-related ultrafine particles and improving patients’ understanding of antibiotic use [[15], [16], [17]]. Ginzberg and colleagues [16] added that visual aids should have minimal and concise text. They also worked with health literacy specialists to help deliver the messages to an audience of varying literacy levels. Van Hecke et al. [16] found that patients were overwhelmed by large quantities of information and ensuring the messages were concise and relevant to the target audience was important. Having a tool, like the infographic, was valuable because it promoted shared decision-making between clinician and patients [17]. Similarly, in our study, we also found that participants valued the toolkit as a method to prompt conversations about climate change and health between providers and patients.

With respect to implementation, two cancer prevention intervention evaluations found that willingness of stakeholders and resources (i.e., time and financial capital) were critical to program success [18,19]. In our study, minimal time with patients due to competing responsibilities was a frequent barrier to implementation. Lazaro et al. [20] found that a multicomponent training for a Tobacco Prevention Toolkit including an information session, website navigation demonstration, and practices helped increase participants’ knowledge of tobacco products and interest in using the prevention resources. This finding aligns with perspectives of some users of the Climate Resilience for Frontline Clinics Toolkit, suggesting that incorporating additional training into future implementations could be beneficial.

### 4.1 Future directions

Our findings support further efforts to develop climate-related education and counseling resources for use in clinical settings but argue for pragmatic approaches that acknowledge time constraints and other challenges. These resources should include actionable information, be designed with the awareness of conflicting demands on clinicians and patients’ literacy level, and be available in multiple languages.

Future research should seek to understand the impacts of implementing these resources on patients’ perceptions, behaviors, and health outcomes, the timing of implementation and strategies to improve implementation. For example, there should be an assessment of how patients perceive the information and whether it leads to actions and improvements in health outcomes. Infographics have been found to be a concise way of delivering patient education on antibiotic use, but formal evidence of effectiveness in climate health education is limited. Furthermore, future studies should seek to identify whether toolkit materials are most effective if employed on a long-term basis as a component of preventative care, or if they are more effective when used on a just-in-time basis when climate-related hazards are forecasted. Lastly, future research on clinic-based climate health education should assess whether additional training for clinicians and staff could improve implementation. Pairing climate education resources with early warning and alert systems and validated screening tools, (e.g. for social determinants of health), to identify at-risk patients may help improve the effectiveness and the efficiency of use.

## 5 Limitations

Since the interviews could only be conducted with participating clinics, who were required to implement the toolkit, we could not capture the feedback of non-implementers. Additionally, there may be response bias since those who were willing to participate in the interview may have had more positive feedback while individuals who did not participate in the interview may have had more negative opinions… Interviewees were from 15 clinics in 6 states, and their experiences may not be representative of the United States as a whole. Generalizability beyond frontline clinics in the United States is limited, given the wide variation in patient needs, healthcare resources and capacity, and relevant hazards in other settings.

## 6 Conclusions

Clinicians and administrators in clinics that implemented the Climate Resilience for Frontline Clinics Toolkit found this resource useful, felt it addressed a previously unmet need, and valued interactions with other clinics. However, they expressed concerns about the amount of information and the need for resources for patients with limited literacy. Information-based toolkits can be valuable to staff in frontline clinics but must be adjusted to meet the specific needs of clinics and the populations they serve.

## Positionality statement

Researchers on this project include students and faculty at the Harvard T.H. Chan School of Public Health as well as staff from Americares. Four identified as Asian or South Asian, and four identified as White. One author is a practicing physician who regularly cares for a wide range of patients, including patients similar to those served by many of the clinic staff interviewed in this study. We bring our diverse backgrounds and experiences to the project and by stating our positionalities hope to add transparency to the work presented.

## CRediT authorship contribution statement

**Yi-Ting Hana Lee:** Writing – review & editing, Writing – original draft, Supervision, Formal analysis, Data curation, Conceptualization. **Mingyue Ma:** Writing – review & editing, Writing – original draft, Formal analysis. **Caroline Sarpy:** Writing – review & editing, Writing – original draft, Formal analysis. **Casey Dai:** Writing – review & editing, Formal analysis. **Jinia Sarkar:** Writing – review & editing, Formal analysis, Data curation. **Chelsea Heberlein:** Writing – review & editing, Resources, Project administration. **Theodore Miles:** Writing – review & editing, Methodology, Conceptualization. **Caleb J. Dresser:** Writing – review & editing, Writing – original draft, Supervision, Investigation, Funding acquisition, Conceptualization.

## Declaration of competing interest

The authors declare the following financial interests/personal relationships which may be considered as potential competing interests: Caleb Dresser reports financial support was provided by Biogen Inc. Caleb Dresser reports a relationship with University of Massachusetts Boston that includes: board membership. Caleb Dresser reports a relationship with Columbia University that includes: consulting or advisory. Caleb Dresser reports a relationship with Johnson & Johnson that includes: funding grants. If there are other authors, they declare that they have no known competing financial interests or personal relationships that could have appeared to influence the work reported in this paper.

## Appendix Supplementary materials

Image, application 1

Image, application 2
