# Supplementary material for: Bringing climate health conversations to frontline clinics: A qualitative post-intervention assessment of utilization of the Climate Resilience for Frontline Clinics Toolkit
Source: J Clim Chang Health. 2025 May 1;23:100444. doi: 10.1016/j.joclim.2025.100444 (PMC12851199; doi:10.1016/j.joclim.2025.100444)
Supplement: Supplementary file 1 [file mmc1.docx]

**Supplementary Table 1. Interview questions with frontline clinics’ clinicians and staff to assess the implementation of Climate Resilience for Frontline Clinics Toolkit**

| Category | Interview Questions |
| --- | --- |
| Context and Impression | 1. Where is your clinic located and what is your role there? 2. Can you tell us how climate-related hazards such as floods, hurricanes, wildfires, and heat waves have affected your clinic this year?    1. How have these hazards affected your patients this year? 3. Why did you choose to try using the Climate Resilience for Clinics toolkit?    1. What were your initial thoughts about the toolkit? |
| Implementation | 1. How did you go about implementing the toolkit?    1. How were other clinic staff members involved in implementing the toolkit? 2. What helped or facilitated your use of the toolkit at your clinic? 3. What were some challenges you and your fellow staff encountered when using the toolkit? 4. How did the staff at your clinics react to the toolkit?    1. Were there any notable or surprising reactions or feedback? 5. How do you connect or communicate with your patients about climate-related hazards like heat, fire, flood, and hurricanes?    1. Were you able to integrate the toolkit into this communication?    2. If so, how did patients feel about the toolkit resources?       1. Any notable or surprising reactions or feedback? |
| Areas for Improvement | 1. What did you find most valuable about the toolkit?    1. Why? 2. What was the biggest problem or issue with the toolkit?    1. What can be done to address this? 3. How can the content in the toolkit be improved to fit your needs? 4. How could the format of the toolkit be improved to fit your needs?    1. Was online PDF format useful?    2. Would you prefer to get your information in another way, for example infographics, videos, flyers, social media, an app, etc.? 5. How can communication with clinics about the toolkit be improved?    1. Would you have liked more orientation or background information?    2. Would you like periodic updates about the content, or reminders when major events such as hurricanes are in the forecast?    3. Would you like a way to hear about what other clinics are doing? 6. If you were to change or add anything to the toolkit, what would that be? 7. Are there complementary resources you’d like to see on this topic?    1. Refrigerator cards with action plans, posters for clinics walls, links to short videos, etc. 8. Please think about the future of your clinic as climate change continues. How do resources such as this toolkit fit into your vision of the future? |
