# Supplementary material for: Bringing climate health conversations to frontline clinics: A qualitative post-intervention assessment of utilization of the Climate Resilience for Frontline Clinics Toolkit
Source: J Clim Chang Health. 2025 May 1;23:100444. doi: 10.1016/j.joclim.2025.100444 (PMC12851199; doi:10.1016/j.joclim.2025.100444)
Supplement: Supplementary file 2 [file mmc2.docx]

We have reviewed the Submission Checklist provided online.

- One author has been designated as the corresponding author and their full contact details (email address, full postal address and phone numbers) have been provided.
- All files have been uploaded, including keywords, figure captions and tables (including a title, description and footnotes) included.
- Spelling and grammar checks have been carried out.
- All references in the article text are cited in the reference list and vice versa.
- Permission has been obtained for the use of any copyrighted material from other sources, including the Web.
- For gold open access articles, all authors understand that they are responsible for payment of the article publishing charge (APC) if the manuscript is accepted. Payment of the APC may be covered by the corresponding author's institution, or the research funder.
